# The power of using functional fMRI on small rodents to study brain pharmacology and disease

**DOI:** 10.3389/fphar.2015.00231

**Published:** 2015-10-21

**Authors:** Elisabeth Jonckers, Disha Shah, Julie Hamaide, Marleen Verhoye, Annemie Van der Linden

**Affiliations:** Bio-Imaging Lab, Department of Biomedical Sciences, University of AntwerpAntwerp, Belgium

**Keywords:** fMRI, rsfMRI, phMRI, BOLD, rodents

## Abstract

Functional magnetic resonance imaging (fMRI) is an excellent tool to study the effect of pharmacological modulations on brain function in a non-invasive and longitudinal manner. We introduce several blood oxygenation level dependent (BOLD) fMRI techniques, including resting state (rsfMRI), stimulus-evoked (st-fMRI), and pharmacological MRI (phMRI). Respectively, these techniques permit the assessment of functional connectivity during rest as well as brain activation triggered by sensory stimulation and/or a pharmacological challenge. The first part of this review describes the physiological basis of BOLD fMRI and the hemodynamic response on which the MRI contrast is based. Specific emphasis goes to possible effects of anesthesia and the animal’s physiological conditions on neural activity and the hemodynamic response. The second part of this review describes applications of the aforementioned techniques in pharmacologically induced, as well as in traumatic and transgenic disease models and illustrates how multiple fMRI methods can be applied successfully to evaluate different aspects of a specific disorder. For example, fMRI techniques can be used to pinpoint the neural substrate of a disease beyond previously defined hypothesis-driven regions-of-interest. In addition, fMRI techniques allow one to dissect how specific modifications (e.g., treatment, lesion etc.) modulate the functioning of specific brain areas (st-fMRI, phMRI) and how functional connectivity (rsfMRI) between several brain regions is affected, both in acute and extended time frames. Furthermore, fMRI techniques can be used to assess/explore the efficacy of novel treatments in depth, both in fundamental research as well as in preclinical settings. In conclusion, by describing several exemplary studies, we aim to highlight the advantages of functional MRI in exploring the acute and long-term effects of pharmacological substances and/or pathology on brain functioning along with several methodological considerations.

## Introduction

Magnetic resonance imaging tools are widely used to evaluate brain structure and function even in a single experiment. The major advantage of MRI techniques is that they are non-invasive, do not use radioactive agents (as opposed to PET) and do not rely on hazardous ionizing radiation (as opposed to CT), rendering MRI a safe imaging tool appropriate for longitudinal follow up. MRI is based on a magnetic field and radiofrequency pulses and most of the MRI applications use the intrinsic tissue contrast relying on different features of ^1^H protons in tissue water without the need of injecting contrast agents.

In short, the technique provides excellent soft tissue contrast, rendering it very appropriate to investigate the brain. Apart from giving anatomical information, MRI allows studying other specific properties of brain tissue using Diffusion-weighted, Diffusion Tensor, and Diffusion Kurtosis Imaging. Additionally, metabolic information can be obtained using Magnetic Resonance Spectroscopy or Chemical Exchange Saturation Transfer. Moreover brain function can be assessed by measuring cerebral perfusion, blood flow (ASL, DSC MRI, and DCE MRI) and brain activity (Functional MRI, rsfMRI, phMRI; [for review see for example (Denic et al., 2011)].

These different techniques can be applied within a single scanning session. After co-registration of the different images, multi-parameter information can be obtained on voxel level or from specific brain regions of interest. Although anatomical and diffusion information allows assessing structural changes induced by neurological disorders, functional changes might occur even much earlier and are of great interest for early diagnosis.

The focus of this review is to describe the use of fMRI to evaluate the effects of pharmacological agents on neuronal activity in small animals using different fMRI techniques. Since its introduction over 20 years ago (Ogawa et al., 1990; Kwong et al., 1992), fMRI has gained immense popularity to study brain activation and brain activity patterns in health (Di Salle et al., 1999; Logothetis, 2008; Bandettini, 2012) and disease (Iannetti and Wise, 2007), both in humans and animal models (Van Der Linden et al., 2007). These methods are especially useful to document the neuro-modulatory actions of pharmacologically active compounds. fMRI allows to determine and localize the target area, that is, the area with the appropriate receptors for the neuromodulator (phMRI). At the same time the technique can estimate the effect on the targeted brain circuitry and potentially beyond (st-fMRI) and rsfMRI. Furthermore, longitudinal fMRI allows to unravel the effect of pharmacological agents upon acute and chronic treatment and one can investigate the interaction between the neuromodulator, the brain and the resulting behavior in the same animal over time.

In this review we try to give an overview of the vast amount of information that can be obtained with small rodent fMRI in pharmacology completed with an overview of specific applications in different animal disease models and their translation to the clinic.

## fMRI Methodology

### Physiological Basis of fMRI

**FIGURE 1:**
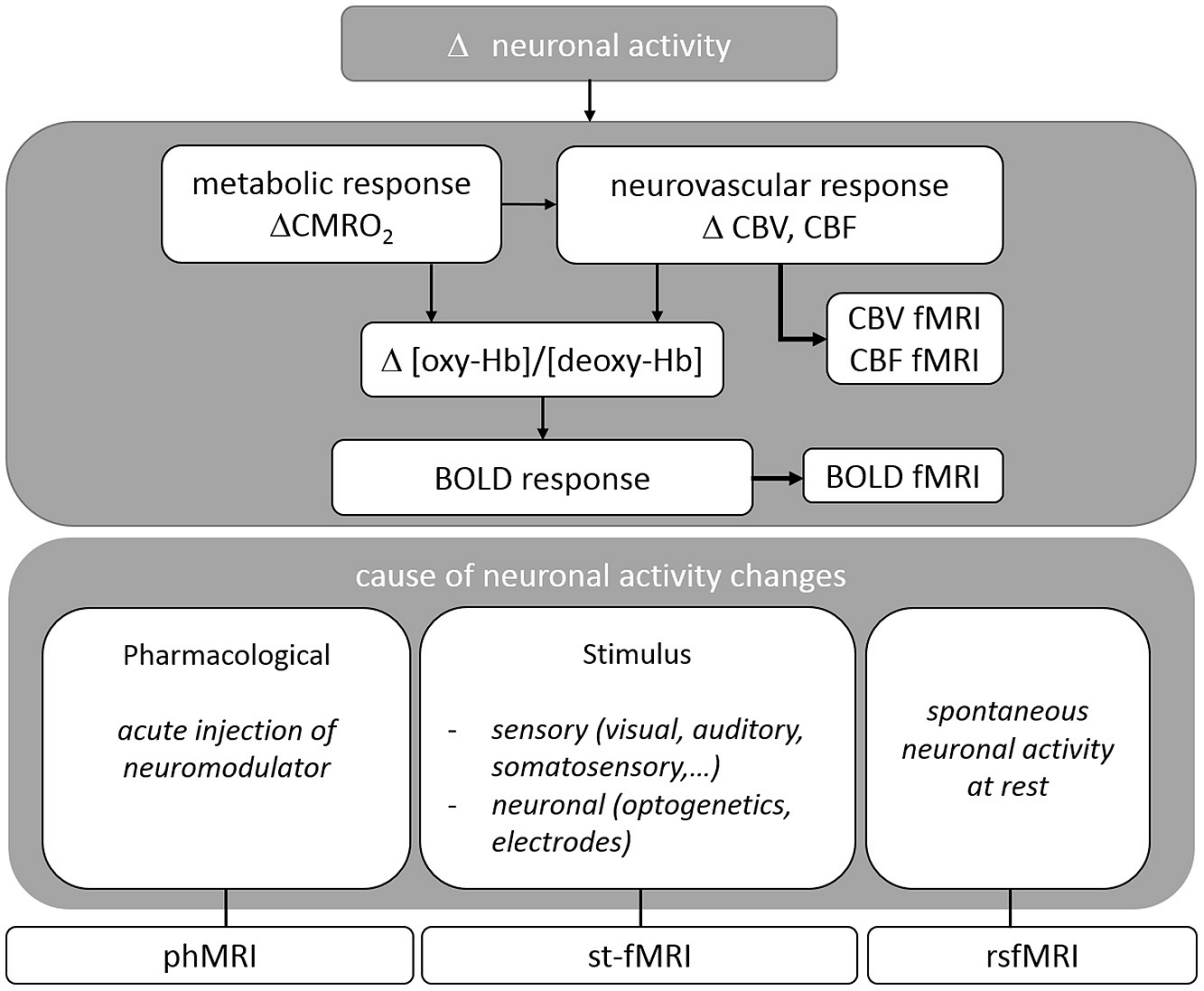
**Overview of the physiological basis of fMRI situating the different techniques reviewed in this paper**.

A variety of MRI pulse sequences exist which exploit different features of water protons in tissue. The most widely used MRI contrasts are found in T1-, T2-, T2^∗^- and proton density weighted images. The resulting images provide superior anatomical contrast allowing qualitative and quantitative assessment of overall brain anatomy. To study brain functioning the sequence is adapted to acquire the BOLD contrast which is based on the differential magnetic properties of oxygenated (diamagnetic) and deoxygenated (paramagnetic) hemoglobin. Upon neural activation, changes in local CBF, CBV, and CMRO2, i.e., the hemodynamic response leads to a locally increased ratio of oxygenated over deoxygenated hemoglobin, resulting in an enhancement in T2^(∗)^-weighted signal intensity (cfr. **Figure 1**). BOLD fMRI is thus an indirect measure of neuronal activity. For a more detailed description please consult (Buxton and Frank, 1997; Logothetis and Wandell, 2004).

The hemodynamic response, is markedly slower (in the order of seconds) than the actual neural activity elicited by neurons, which is in the order of milliseconds (Logothetis et al., 2001; Logothetis and Wandell, 2004). Although over recent years, numerous variations to the well-established EPI and GRASE pulse sequences have been developed enabling a repetition time up to 0.500 s [for review of recently implemented fast and high-resolution fMRI sequences (Feinberg and Yacoub, 2012)] this strongly contrasts to other functional imaging measurements such as EEG, MEG, NIRS which provide more direct information and require a substantially higher temporal resolution (sampling frequency in the range of kHz). Despite its relatively low temporal resolution, fMRI provides the best three dimensional spatial resolution covering the entire brain in comparison to the other techniques mentioned above.

Magnetic resonance imaging also allows to detect brain activity based on local changes in CBF or CBV and specific methods exist to assess these changes. One of these methods is ASL, in which the inflowing water proton spins in the arterial blood are magnetically tagged (inverted) serving as endogenous tracer to observe the effects of inversion on the contrast of brain MRI which can then be used to measure stimulus-evoked changes in CBF (Williams et al., 1992; Detre and Wang, 2002).

In CBV-weighted fMRI (Mandeville et al., 1998; Smirnakis et al., 2007; Ciris et al., 2014), injection of exogenous intravascular contrast agent (e.g., iron oxide particles) is required to monitor brain activity changes represented by signal intensity changes induced by local CBV alterations (Mandeville, 2012).

Within this review we opted to describe BOLD based fMRI techniques and applications while methods using labeling methods or contrast injections -which are also applied in pharmacological research (Borsook and Becerra, 2011; Jenkins, 2012)- were considered beyond the current scope.

### Different fMRI Methods

Different applications of BOLD fMRI allow for the study of neuronal activity after imposing a certain task or stimulus (**st-fMRI**), during rest (**rsfMRI**), or as response to acute drug challenges (**phMRI**). In the following sections, each of these techniques will be discussed in detail (For an overview cfr. **Figure 1**).

**Stimulus-evoked fMRI** allows for the investigation of neural activity as a response to a specific stimulus or task. In most rodent st-fMRI studies externally applied sensory-motor stimuli are used while in human st-fMRI studies also cognitive task can be applied. The most commonly used stimulus presentation designs for fMRI experiments are (i) a block design and (ii) an event-related design (Amaro and Barker, 2006). In a block design, two or more different conditions are alternated in order to determine the differences between the two conditions. A control or rest situation may be included in the presentation occurring between the two conditions. In event related designs the time in between stimuli can vary while also much shorter stimuli are used. Although preclinical studies most often implement block designs, event-related designs are becoming more and more established (Schlegel et al., 2015).

Instead of using sensory or cognitive stimuli, BOLD fMRI can be used to study the direct effect of pharmacological modulations on neuronal activity (**phMRI**). In this case the acute injection of a compound during the fMRI scan evokes changes in the BOLD response in brain areas that express specific receptors for the injected compound and also in their projection areas. In phMRI the experimental design is dependent on the pharmacokinetic and pharmacodynamic profile of the drug. The inability to control, or even to know the timing and amplitude of the stimulus renders this method considerably more challenging than conventional st-fMRI studies where the timing depends on the predefined paradigm (Jenkins, 2012).

Both techniques, fMRI and phMRI, can be combined when investigating the modulatory effects of a pharmaceutical compound on a conventional st-fMRI read-out, such as the effects of dopaminergic drugs on cognitive tasks (Dodds et al., 2009). Moreover, repeated phMRI studies after chronic drug application could provide insights in the resulting adaptations of the brain such as changes in receptor density or sensitivity. However, additional measures (such as CBV and CBF) are required in case the used pharmacological compound has concomitant vaso-active properties.

Functional imaging can also be used to study FC by monitoring BOLD signals at rest (**rsfMRI**). During rest, it was shown that the BOLD signal shows spontaneous fluctuations over time. Functional connectivity is defined as the temporal correlation of low frequency (0.01–0.1 Hz) fluctuations of the BOLD signal between spatially distinct brain regions (Lowe et al., 2000). Consequently, the spontaneous low frequency fluctuations of the BOLD signal are used as indirect marker to depict the functional architecture of the brain.

Several applications of rsfMRI have demonstrated that the healthy brain is organized into functional networks and that these networks can be affected by neurological disorders. In humans, and to some extent also in rodents, large scale ‘resting state networks’ can be detected that include brain regions involved in auditory processing, motor function, visual processing, memory, and executive functioning (Damoiseaux et al., 2006; Jonckers et al., 2011). Moreover networks anatomically homologous to the so-called human ‘default-mode network’ (DMN), which is activated during rest and deactivated during goal-directed tasks (Fransson, 2005; Upadhyay et al., 2011; Lu et al., 2012; Sierakowiak et al., 2015) and its anti-correlated ‘salience network’ (SN) are also detected in rodents (Sforazzini et al., 2014).

### fMRI Data Processing and Presentation

Although most fMRI processing software so far was optimized for human data, processing strategies are usually similar in humans and rodents. Also, an increasing number of analysis packages are being tailored to fMRI studies of rodents (Sawiak et al., 2009; Chavarrias et al., 2015).

Functional MRI data (fMRI, rsfMRI, and phMRI) are typically pre-processed before the actual data analysis. The most commonly used software packages are SPM^1^ and FSL^2^ Pre-processing includes (1) slice timing correction and realignment over time, (2) spatial normalization to a standardized stereotactic space, and (3) smoothing (James et al., 2014). An important difference between human and rodent fMRI processing is the way group data are handled. The Montreal Neurological institute (MNI) defined a standard brain from a large series of MRI scans of normal controls (Evans et al., 1992). For instance, this template is automatically provided in SPM, one of the commonly used fMRI processing toolboxes. Although the same toolbox can be used for rodent fMRI, the rodent data has to be normalized to a study-specific template such as the mean of all control animals. Alternatively, an atlas can be developed from in-house measurements or obtained from a publically available source [e.g., (mice)^3^; (mice, rats, etc.^4^).

Resting state fMRI data pre-processing might include extra steps such as GS regression, and temporal filtering. GS regression serves to remove global fluctuations that mask circuit-level organization, to remove global physiological artifacts, and to enhance the reliability of the experimental results (Fox et al., 2009). Temporal filtering may be included to restrict to fluctuations between 0.01 and 0.1 Hz, which are of interest in rsfMRI research. Nevertheless, recently an increasing number of studies explored correlations in BOLD fluctuations beyond this frequency band. The resulting data suggested that long-distance connections peak at low frequency bands, whereas short-distance connections are distributed in a relatively wider frequency range. Moreover, the dominance of different frequencies seems to characterize different brain networks. (Wu et al., 2008; Boubela et al., 2013).

After pre-processing, st-fMRI and phMRI data can be processed by different methods. Typically, a voxel-based approach is adopted. After pre-processing, the BOLD signal is modeled (e.g., general linear model) as the convolution of the applied stimulation design (block or event-related design) with the hemodynamic response function (HRF) in order to have a better estimation of the true design-related BOLD signal (Lindquist et al., 2009).

Finally, statistics can be performed on the model estimates (i.e., stimulus specific BOLD responses) via parametric or non-parametric methods resulting in statistical maps indicating areas of activation. These maps are first created on the single subject level (first level analysis) after which they are used in second level analyses to perform group statistics. To define brain regions activated by acute compound injection (phMRI) also a voxel based approach can be applied by statistical comparison of the repetitions before injection (baseline) with those after injection.

**FIGURE 2:**
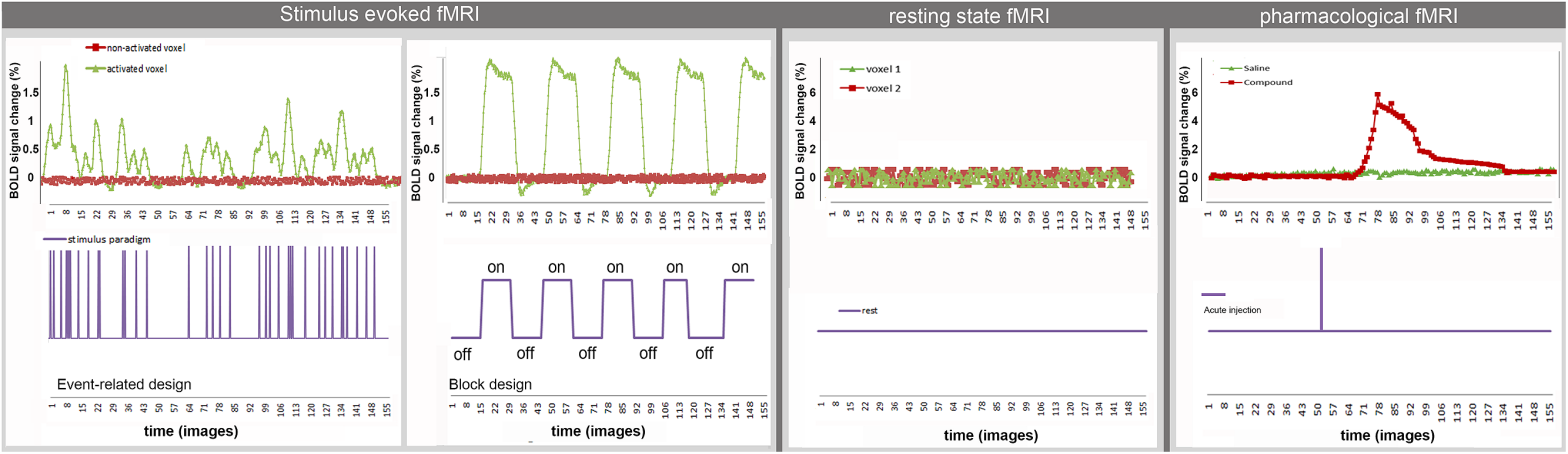
**Example of typical time courses for st-fMRI, rsfMRI, and phMRI**.

The aforementioned analyses techniques do not take into account the temporal dynamics of the BOLD-signal after sensory or pharmacological stimulation. Upcoming analysis methods apply spectral analysis to the BOLD time series to obtain information on the temporal behavior of the BOLD response function [for more details see (Muller et al., 2001)]. **Figure 2** shows examples of typical time courses for the different techniques and how they are related to the applied stimulus. Both rsfMRI and event related st-fMRI need fast MRI sequences (e.g., TR = 2 s, i.e., one image in 2 s) to be able to acquire BOLD fluctuations and responses to the fast consecutive stimuli, respectively. A typical rsfMRI acquisition lasts 5–12 min. In a block design the length of the different blocks in the paradigm determines the required acquisition speed. Moreover the total scan-length is dependent on the complexity of the paradigm, increasing the number of scans needed when more stimuli are introduced or the differences between stimuli are more subtle. For phMRI the timing is dependent on the pharmacokinetics of the injected compound.

**FIGURE 3:**
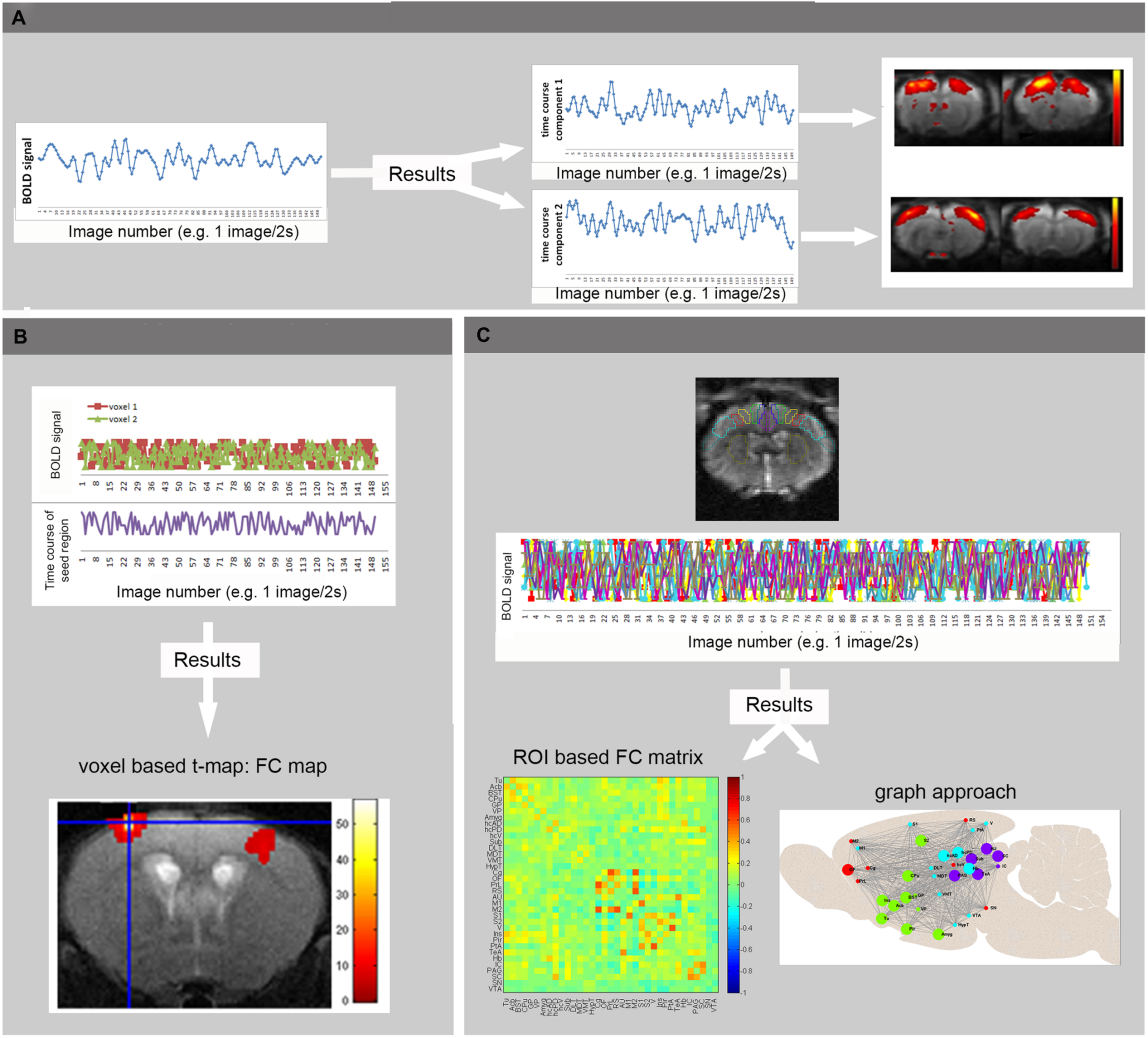
**Basic principles of rsfMRI Analysis. (A)** Independent Component Analysis divides the BOLD signal of all brain-voxels in different spatially confined independent sources, or components. Each ICA component consists of brain regions with correlated BOLD time courses. In other words, voxels of one component represent regions that are functionally connected. **(B)** In voxel-based analyses, the mean BOLD signal time course of a specific seed region is extracted from a series of EPI images. This time course is compared to the time course of all other voxels in the brain, resulting in a functional connectivity map (voxel based processing). **(C)** In an ROI based approach, the mean BOLD signal time courses of multiple brain regions are compared, resulting in FC matrices showing the strength of connectivity between each pair of brain regions (warmer colors indicate stronger functional connectivity, colder colors represent anti-correlation). These matrices can then be used to visualize brain networks as nodes (brain regions) and edges (connections). Moreover, the brain network can be divided into modules that represent brain circuits where similar time courses are displayed by different colors (graph approach). (Adopted from Jonckers et al., 2013b).

For processing of **rsfMRI** data, various software packages exist, supporting different processing strategies (Margulies et al., 2010). The most widely used methods in FC analysis of resting state data are ROI-based (Biswal et al., 1995), seed-based and model-free, data-driven approaches such as ICA (Calhoun et al., 2001; Beckmann and Smith, 2004). Other data-driven techniques are clustering approaches and graph analysis [for methodological review: (Margulies et al., 2010)] (see **Figure 3**). Finally alternative processing techniques are available to map the directionality of the connectivity, such as dynamic causal modeling (Friston et al., 2003) and Granger causality analysis (Roebroeck et al., 2005) which can also be applied on activity-induced fMRI data to define regions that drive the activation. Apart from FC analysis, also the low frequency fluctuation themselves can be affected during disease conditions and studied using ALFF analysis. ALFF is defined as the total power within the frequency range between 0.01 and 0.1 Hz, and thus indexes the strength or intensity of Low Frequency Fluctuations. Measurements of ALFF are more often applied in humans but some rodent students already report changes in ALFF in animal models (Li et al., 2012; Yao et al., 2012).

### Important Considerations when Planning fMRI Experiments in Rodents

Rodent fMRI studies have great potentials and can provide an immense contribution to pharmacological research, but it must be underscored that attention and expertise is necessary when rodent fMRI experiments are designed and performed. The functional status of the brain is highly dependent on the physiology of the animal. Much more than for anatomical imaging, monitoring and controlling for this physiology is essential during rodent fMRI studies. Accounting for physiological changes is especially important in rodent studies since typically animals need to be anesthetized.

Moreover optimized protocols of the imaging set-up and processing tools for rats can not always simply be duplicated for mice since there are important differences between these rodent species, which we outline below.

First of all, it is important to mention that most of the reported fMRI work in rodents is performed in rats, despite the existence of a wide array of mouse models mimicking neurological disorders. One of the reasons behind this is the fact that it is very challenging to acquire reproducible brain activation upon stimulation in mice. Since the introduction of rsfMRI, more studies use mice as an animal model but most of the basic research, (i.e., optimization of analysis and unraveling the underlying mechanisms) is still performed in rats. Nevertheless, it is very important to take into account that conclusions drawn in rats are not necessarily applicable in mice. Moreover, recent advances such as the development of cryo-coils have dramatically improved mouse fMRI (Ratering et al., 2008).

When studying functional connectivity using rsfMRI, one should also consider the impact of hormones and age-related hormonal changes on brain connectivity as was demonstrated in human studies. In a recent review, the association between functional connectivity and endogenous sex hormone fluctuations across the menstrual cycle in humans was described (Weis et al., 2011). When endogenous estradiol and progesterone levels are high, functional communication between both hemispheres is enhanced. Also gender and overall hormonal status throughout the lifespan of an individual has a major impact on the functional connectivity of the brain. Specifically, ovarian hormones (estradiol and progesterone) may enhance both cortico-cortical and subcortico-cortical functional connectivity, whereas androgens (testosterone) may decrease subcortico-cortical functional connectivity but increase functional connectivity between subcortical brain areas. Similar investigations using rsfMRI in rodents have not been reported yet but human studies suggest that caution is required when examining healthy brain development and aging or when investigating possible biological mechanisms of ‘brain connectivity’ diseases. Therefore, the contribution of sex steroids should not be ignored (Peper et al., 2011).

#### Effects of Anesthesia on the st-fMRI and phMRI Outcome

**FIGURE 4:**
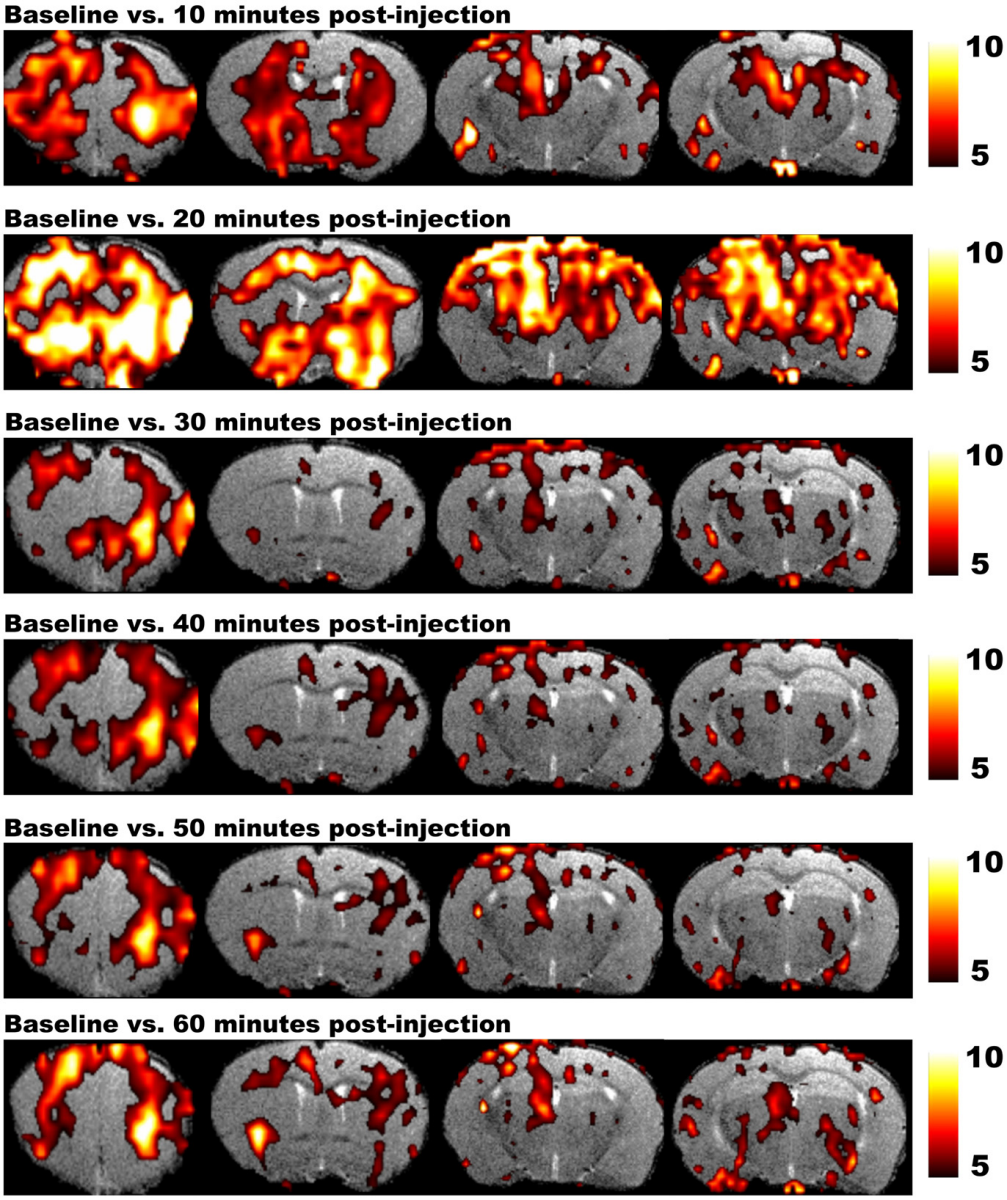
**Results of phMRI with a single bolus of medetomidine.** This figure shows four consecutive slices of the statistical difference maps of the BOLD signal at 10, 20, 30, 40, 50, and 60 min post-injection (medetomidine, 0.3 mg/kg, s.c.) vs. baseline. The statistical maps are shown on a T2-weighted anatomical MRI template. The color scale at the right indicates the *T*-value (i.e., the strength of the T2^∗^ signal intensity decrease induced by medetomidine vs. baseline in all conditions). Medetomidine induces T2^∗^ signal changes at 10 min post-injection vs. baseline, mainly in frontal regions and the striatum. At 20 min post-injection, the T2^∗^ signal changes increase and include additional regions such as the sensory cortex, hippocampus and thalamus. Starting from 30 min post-injection, medetomidine-induced T2^∗^ signal changes start to decrease and stabilize until 60 min post-injection (Shah et al., 2015a).

Magnetic resonance imaging studies in rodents require the use of anesthetic agents to minimize stress and to prevent motion artifacts during the scans. Several anesthetics are optimized for MRI acquisitions, but for fMRI it is utterly important to take into account how the anesthesia affects neuronal activity and the hemodynamic response. Moreover, distinct levels of consciousness could result in different fMRI outcome. Finally, when a pharmacological agent is used in phMRI, possible interactions between the pharmacological compound and the applied anesthetic have to be taken into account as well (Hodkinson et al., 2012). On the other hand phMRI protocols can be used to assess the time-dependent effects of anesthetics on the BOLD signal. **Figure 4** shows the T2^∗^ signal intensity changes over time induced by a single bolus of medetomidine (Shah et al., 2015a). These changes over time underscore the need for a highly optimized anesthesia protocol applied in exactly the same manner to every animal within the study (Magnuson et al., 2014). Moreover stable conditions can be obtained by combining bolus injections to induce anesthesia followed by continuous infusion.

A robust and reproducible BOLD response can be observed in rats and mice anesthetized with commonly used anesthetics for MRI, such as medetomidine, isoflurane, α-chloralose and urethane (Austin et al., 2005; Adamczak et al., 2010). Nevertheless, under anesthesia, more intense stimuli must be presented to evoke a BOLD response in comparison to awake conditions. The cerebral hemodynamic response upon sensory stimulation shows an anesthesia-specific modulation which can be largely explained by the effects of the anesthetics on animal physiology. Strikingly, independent of the anesthetic used, fMRI responses may additionally be influenced by stimulus-induced cardiovascular changes, which may mask specific fMRI signals associated to the stimulus (Schroeter et al., 2014).

The anesthetic agent **α-chloralose** is a GABA-A agonist that binds to a site on the GABA-A receptor complex distinct from the benzodiazepine neurosteroid and barbiturate sites (Garrett and Gan, 1998). It has long been considered the gold standard for fMRI due to its minor effects on cardiovascular function, and is presumed to only minimally suppress neuronal activation. When using α-chloralose, strong neuronal activation is induced even with very subtle stimuli such as whisker deflection. Nevertheless, due to its toxicity -mainly for the liver- it has been considered as a terminal drug and its use was abandoned.

However, it was recently demonstrated that using a new formulation in a careful application scheme this anesthetic allows for repeated fMRI studies on the same rat (Alonso Bde et al., 2011).

**Medetomidine,** which results in sedation rather than deep anesthesia, is the anesthetic of choice for longitudinal functional imaging studies in rats (Fukuda et al., 2013). Medetomidine is an α2 agonist predominantly acting on presynaptic receptors in the locus coeruleus, resulting in decreased noradrenaline release. Medetomidine typically induces cerebral vasoconstriction mediated by direct agonist binding to receptors on the cerebral vessels resulting in reduced baseline CBV and CBF (Nakai et al., 1986). The degree of vasoconstriction depends on the dose and delivery method (topical vs. systemic). The effects of medetomidine can easily be reverted with Atipamezole (Scheinin et al., 1987).

Inhalation anesthetics (e.g., **isoflurane**) are preferred for longitudinal fMRI experiments (Kim et al., 2010) due to fast recovery and low mortality rates. Isoflurane acts on γ-amino butyric acid type A (GABA-A) receptors through depression of excitatory synaptic transmission (Larsen and Langmoen, 1998). Importantly, most inhalation anesthetics evoke vasodilation in a dose-dependent manner, which might obscure the actual experimental outcome since changes in vascular properties are very likely to affect the BOLD signal. Therefore, functional experiments are typically performed under lower doses (1–1.5%) as compared to structural MRI studies (2%). The dose used can even be lowered by combining isoflurane with medetomidine. This combination protocol has the additional advantage that the epileptic activity, seen when medetomidine is infused for longer than 120 min, is prevented, enabling longer experiments with equally fast recovery than for medetomidine alone (Fukuda et al., 2013).

Finally, **propofol and urethane** anesthesia, which both enhance GABAergic transmission, are occasionally used for fMRI experiments, in comparison to other previously described anesthetics, the induced fMRI activation is lower under the same stimulus intensity which impedes the detection of very subtle functional alterations (Lahti et al., 1999; Huttunen et al., 2008).

#### Effect of Anesthesia on Functional Connectivity as Assessed by rsfMRI

Apart from affecting the BOLD response post-stimulus, anesthetic agents could influence the correlations in intrinsic activity used to estimate functional connectivity. Moreover, these effects are different for rats and mice.

**Medetomidine** is considered as the gold standard for FC mapping in rats based on its reliable and spatially specific outcome (Kalthoff et al., 2013). In mice, however, medetomidine induces decreased inter-hemispheric FC (Jonckers et al., 2011) resulting in the need of other anesthesia protocols in these animals (Jonckers et al., 2013a). Important dosage effects showing decreased inter-hemispheric connectivity in the rat brain at high dose might explain the decreased inter-hemispheric FC in mice, which require a relatively higher dose compared to rats (Nasrallah et al., 2014).

Rats anesthetized with **isoflurane** show less localized clusters of high FC unless a low dose of 1% is used (Williams et al., 2010). In mice, however, it is not straightforward to gain consistent results using the same dose of isoflurane as in rats creating the need for adapted protocols (Jonckers et al., 2013a). Interestingly, combining a low dose of medetomidine with a low dose of isoflurane seems to give the required results (Grandjean et al., 2014a).

**α-chloralose and urethane** also allow robust detection of FC in rats (Hutchison et al., 2010; Williams et al., 2010; Jonckers et al., 2011) suggesting that the strongest connections are preserved even during deeper states of anesthesia (Bettinardi et al., 2015). Besides physiological confounds, changes in resting-state networks may reflect a functional reorganization of the brain at different anesthesia levels or brain states related to the level of consciousness (Liu et al., 2013). For example **Propofol** shows a dose-dependent decrease of thalamo-cortical FC (Tu et al., 2011; Liu et al., 2013).

Although the sensori-motor networks are detected with the different anesthesia regimes mentioned above, lower doses seem to be needed to preserve the DMN-like and SN. This effect may infer that these networks support higher level consciousness since these networks are well defined in awake rats (Upadhyay et al., 2011). Using a low dose of isoflurane, both the DMN-like and SNs could be detected in mice (Sforazzini et al., 2014; Liska et al., 2015). The long range anterior–posterior connections in the DMN-like network seem to be disturbed after medetomidine injection but restored when the level of anesthesia is lowering over time (Shah et al., 2015b).

The aforementioned effects of anesthesia on fMRI results yielded several attempts to optimize awake imaging protocols (Ferris et al., 2011). Moreover, weak functional connections are more likely to be picked up in awake animals (Liang et al., 2012). The drawback to awake imaging, however, is that the brain may be in different functional states depending on how well the conscious animals are acclimatized to the MRI scanner environment (Upadhyay et al., 2011) and this variance in consciousness will contribute to an increased variability in the fMRI outcome.

Nevertheless, several groups succeeded in optimizing training protocols, showing that rats can adapt to the scanner environment (King et al., 2005) resulting in reliable fMRI results (Febo, 2011) and robust reproducible resting state networks (Zhang et al., 2010; Becerra et al., 2011).

Mice, however, seem to be more difficult to train for these types of experiments (Jonckers et al., 2013a). The labor intensive effort of training protocols is not always suitable in experiments with high numbers of animals (e.g., when different groups are compared) or when following animals over time, especially starting from a very young age. Therefore, the application of awake imaging in models of neurological disease will remain limited.

#### The Need for Monitoring and Controlling Physiological Parameters

Since fMRI measures signals related to the hemodynamic response, a stable physiology of the animal during fMRI acquisitions is of uttermost importance to enable accurate and reproducible measurements. Therefore, physiological parameters need to be monitored and if possible continuously adjusted.

**Partial pressure of CO_**2**_** (pCO_2_) must be monitored during the fMRI measurements for two major reasons. First, increased pCO_2_ leads to vasodilatation and thus an increased CBV. Increased CBV results in a reduced stimulus-induced hemodynamic response. Second, increased pCO_2_ leads to a decreased oxygen affinity of Hb (the so-called Bohr effect), changing the ratio of oxygenated over deoxygenated hemoglobin. Both phenomena affect the BOLD signal without any underlying neuronal origin. In the past, pCO_2_ monitoring was achieved through repetitive blood sampling. Currently, continuous and non-invasive recordings of transcutaneous pCO_2_ (Mueggler et al., 2001) or end-tidal pCO_2_ values with MRI-compatible capnometry are used (Silverman and Muir, 1993; Van Camp et al., 2005) for which linear correlations with arterial pCO_2_ have been established (Zhang et al., 1997).

**Body temperature** is the second major modulator, as the BOLD signal shows a strong negative correlation with body temperature due to a decreased oxygen affinity of Hb with increasing temperature (Hyder et al., 1994).

Moreover, the brain’s metabolism is also affected by the body temperature of the animal. Consequently, temperature changes can mask the true contribution of neuronal activity (Vanhoutte et al., 2006). Since the body temperature gradually lowers in anesthetized rodents, a feed-back mechanism with hot air or warm water circuit is vital to keep the temperature at ±37°C.

Apart from the aforementioned parameters, follow-up of **blood pressure, heart rate,** and **breathing rate** are essential as a read-out of the animal’s sedative state and will affect the hemodynamic response. Typical values for the different parameters are highly dependent on the anesthesia protocol and differ between rats and mice. For example the heart rate in awake rats is about 400 beats per minute in comparison to 600 beats per minute in mice. Also breathing rate is typically lower in rats (±85 breaths per minute) than in mice (±150 breaths per minute) making mouse fMRI more dependent on motion artifacts due to breathing. Especially during the fMRI acquisition it is essential to gain a stable physiology to induce as less variation as possible. (Bernstein, 1966; Baker et al., 1979).

A better control of blood gas parameters and breathing rate can be obtained by mechanical ventilation. Gated imaging, in which the imaging sequence is triggered by the respiratory cycle, can then be used to reduce motion artifacts caused by breathing (Cassidy et al., 2004). New methods have been suggested that allow recognition of artifacts and subsequent removal from the fMRI data (Salimi-Khorshidi et al., 2014).

Finally, when acute drug effects are investigated, it is important to take into account the systemic effect of the drug on the physiology of the animal which in turn could influence the BOLD signal. For example, pharmacological induction of vasodilation with acetazolamide attenuates the activity-induced BOLD response resulting from an increase in CBF (Bruhn et al., 1994).

### The Advantage of Hybrid Systems: Simultaneous PET/MRI and MRI/Electrophysiology

Apart from fMRI, PET studies can be used to assess brain activation using the short half-life tracer [(15)O]H_2_O as a marker for CBF or the radiotracer FDG [(18)F]FDG which estimates glucose metabolism. Higher sensitivity of PET, combined with a better contrast-to-noise ratio and spatial resolution for BOLD fMRI underlies the rationale for combining both techniques.

Interestingly, resting state networks can be defined using both PET and rsfMRI. Consequently, simultaneous PET/MRI systems are gaining more and more interest (Wehrl et al., 2014), since simultaneous studies can reveal/provide comprehensive and complementary information to further decode brain function and brain networks (Wehrl et al., 2013).

Combining electrophysiology with fMRI allows for the correlation of indirect BOLD signals with the underlying neurological ones (Logothetis, 2002; Logothetis and Wandell, 2004; Sloan et al., 2010; Sumiyoshi et al., 2012). Evoked LFP measured simultaneously with the BOLD response (Huttunen et al., 2011) show the neuronal origin of the spontaneous BOLD signal measured during rest. The low frequency fluctuations in the BOLD signal are significantly correlated with infra-slow LFP signals as well as with the slow power modulations of higher-frequency LFPs (1–100Hz) at a delay comparable to the hemodynamic response time under anesthesia (Pan et al., 2013). Nevertheless, the combination of MRI and electrical recording is technically challenging because the electrodes used for recording need to be MRI compatible and the MRI acquisition induces noise in the electrical recording. To minimize the mutual interference of the two modalities, glass rather than metal microelectrodes can be used and noise removal algorithms are implemented to analyze electrophysiology data (Pan et al., 2010).

## fMRI Applications with Relevance for Pharmacological Research

The non-invasive nature and possibility to translate preclinical findings to the clinic render the multiple fMRI techniques outlined in this review into attractive methods for a wide variety of pharmacological applications. Indeed, phMRI can be used to unravel underlying neurobiological mechanisms of drug action and neurotransmitter-related disorders (Canese et al., 2011). Moreover, phMRI enables the investigation of a specific neurotransmitter system after administering known compounds, e.g., investigation of dopamine D2 transmission after dopamine reuptake inhibition (Squillace et al., 2014).

A very specific approach was proposed by Schwarz et al. (2007a,b) based on a combination of phMRI and FC analysis of rsfMRI data. The outcome convincingly identified connectivity patterns underlying the central effects of the injected compound. This approach can be extended by modulating the FC in an antagonist–agonist framework. First, a certain connectivity pattern is induced by acute injection of a first known compound during the scanning session. Next, the second compound of interest is also injected during the same scanning session inducing a modulation of this known connectivity pattern (Schwarz et al., 2007c; Shah et al., 2015b).

**FIGURE 5:**
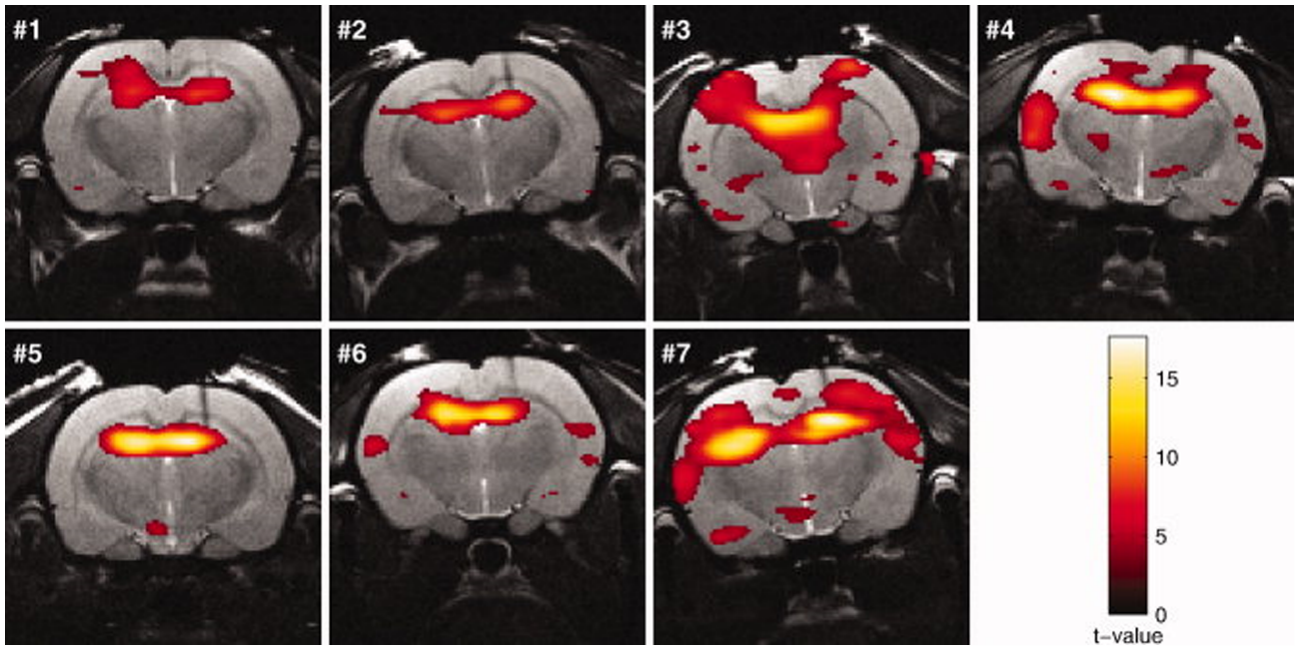
**Activation maps (rats no. 1–7) in response to kainic acid–induced seizures superimposed on the anatomic images.** The threshold for statistical significance was set at *p* < 0.05 (FWE corrected). Rats were sedated with medetomidine (figure reproduced with permission of John Wiley and Sons; Airaksinen et al., 2010).

The added value of fMRI depends on the pathological phenotype of the disorder and the most prevalent pathologies will be discussed in detail below. The following sections will discuss how different fMRI techniques have led to important insights into several of the most prevalent pathologies. For example, in neurodegenerative disorders changes in brain function potentially precede structural degradation. Both st-fMRI and rsfMRI can be used to study neurological changes on a functional level, which might be of interest in terms of early diagnosis and drug intervention before the occurrence of irreversible damage. During and after therapeutic interventions the same techniques can be used to determine the efficacy of a treatment (both acutely and longitudinally) and assess recovery of functional networks (see **Figure 5** for an overview).

### (rs)fMRI Studies in Pharmacologically Induced Models

Based on the NMDA depletion theory in **schizophrenia,** NMDA receptor antagonists (e.g., memantine) are used to mimic this disorder in rodents. Typically, behavioral assessments are generally accepted as a reliable read-out and display locomotor hyperactivity after NMDA receptor antagonist administration. Multimodal MRI extends the characterization of this model showing dose-dependent pharmacological activation in the prelimbic cortex after acute memantine administration. Sub-chronic memantine injection revealed significant effects in the hippocampus, cingulate, prelimbic, and retrosplenial cortices. These are potentially vulnerable regions in schizophrenia and are known to be involved in the mediation of specific cognitive functions affected in schizophrenia (Tamminga, 2006).

Interestingly, FC as well as ultra-structural features, defined with diffusion imaging, were significantly decreased in the same regions (Sekar et al., 2010). Similar FC results were reported in a genetic model for schizophrenia (Song et al., 2015).

Additionally, the effects of new antipsychotic drugs can be tested using the same read-outs, enabling target validation and early assessment of drugs (Sekar et al., 2010; Bifone and Gozzi, 2012; Song et al., 2015).

**Seizures** are due to abnormal, excessive, or synchronous neuronal activity in the brain. They can be pharmacologically induced in rats by increasing neuronal excitation, for example by excessively activating glutamate receptors (kainic acid) or acetylcholine receptors (pilocarpine) or by decreasing inhibition with antagonizing GABA-A (bicuculline; Fritsch et al., 2014).

**FIGURE 6:**
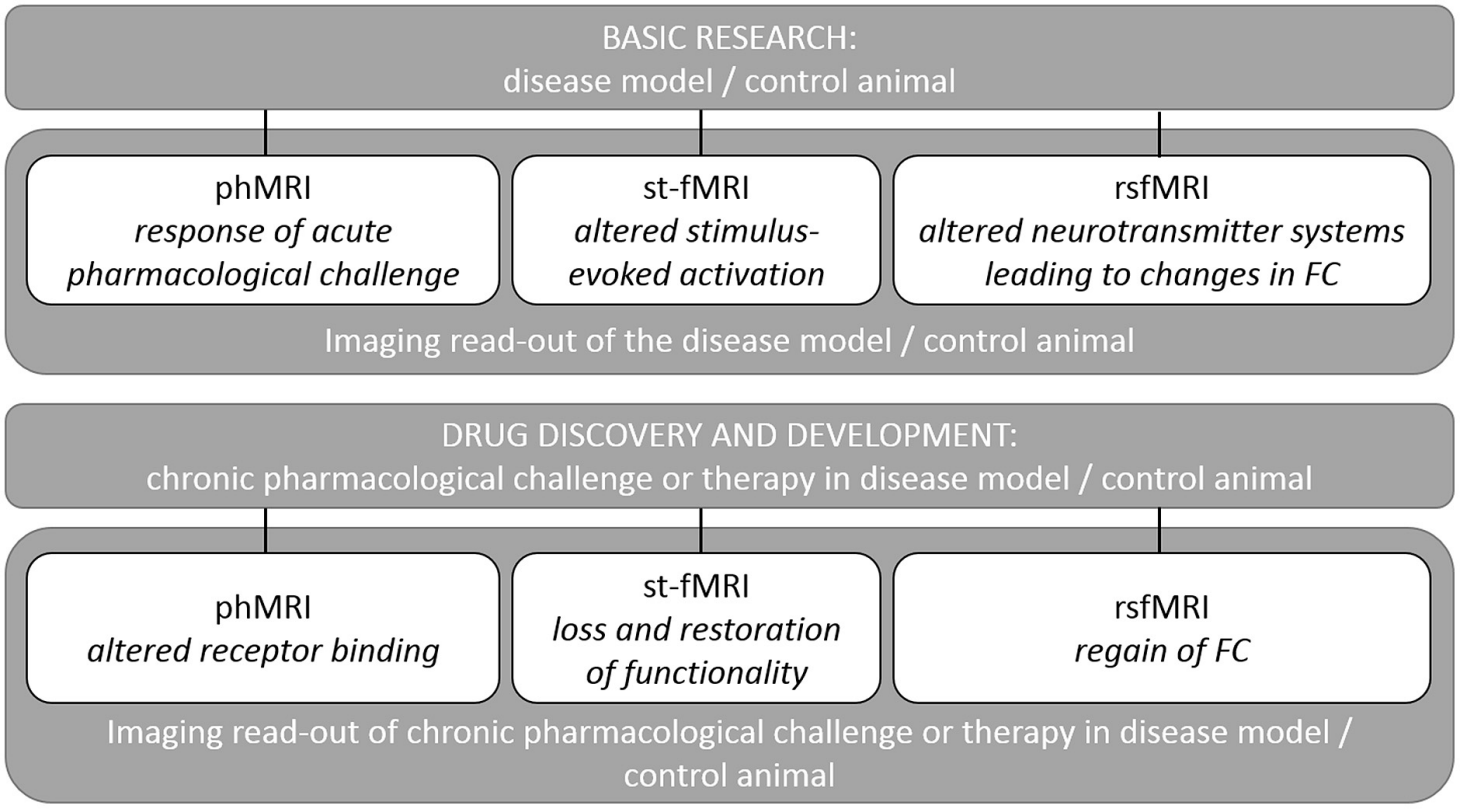
**Overview of the applications of fMRI methods in research explaining the outcome for each type of fMRI acquisition.** Detailed examples are given in the text.

For example systemic kainic acid injection induces limbic seizures originating from the hippocampus (Ben-Ari et al., 1981) which can be monitored with st-fMRI (Airaksinen et al., 2010; DeSalvo et al., 2010; see **Figure 6**). Moreover, BOLD activation as a response to electrical stimulation is modulated upon consecutive seizures (Vuong et al., 2011).

Simultaneous BOLD and electrophysiological recordings show potential decoupling of the BOLD response from neuronal activity in a small number of seizures defined with electrophysiology. This may relate to a development of recurrent seizure activity (*status epilepticus*), which associates with remarkably increased cerebral metabolic rate of CMRO2. If CMRO2 reaches the limit of the compensatory capacity of CBF, no positive BOLD response can be detected which has implications for the interpretation of st-fMRI data obtained during prolonged epileptiform activity (Airaksinen et al., 2012). RsfMRI can be used to explore how seizures modulate the affected brain circuitry. This rsfMRI functionality was shown in (WAG/Rij) rats –a model for human absence seizures– where researchers reported increased cortico-cortical correlations in rest in comparison to non-epileptic controls. This finding is indicative of augmented FC between brain regions which are most intensely involved in seizures (Mishra et al., 2013).

Similar to Major Depression Disorder (MDD) in humans, and using rsfMRI, the inbred Wistar Kyoto More Immobile rat, an accepted model for depression, shows functional connectivity anomalies between hippocampus, cortical, and sub-cortical regions (Williams et al., 2014). Serotonin 1A receptor (5-HT1A-R) knockout mice or healthy mice administered a specific 5-HT1A-R antagonist have been used to mimic brain serotonin depletion in **depression.** Both show reduced FC of the serotonergic system (Razoux et al., 2013). Moreover, st-fMRI can be used to assess the neural substrate of typical MDD behavioral characteristics. Changed BOLD responses to fear stimuli in the cortico-amygdalar network as well as the insular cortex may be the basis for fear and aversion in depression (Huang et al., 2011).

Treatment of depression disorders consists of increasing the level of 5HT, for example with SSRI. The resulting acute activation of the 5-HT system can be picked up with phMRI (Sekar et al., 2011a,b; Klomp et al., 2012a). Interestingly, phMRI studies show that the response to acute SSRI challenge changes in chronically SSRI treated adult animals (Klomp et al., 2012b).

**FIGURE 7:**
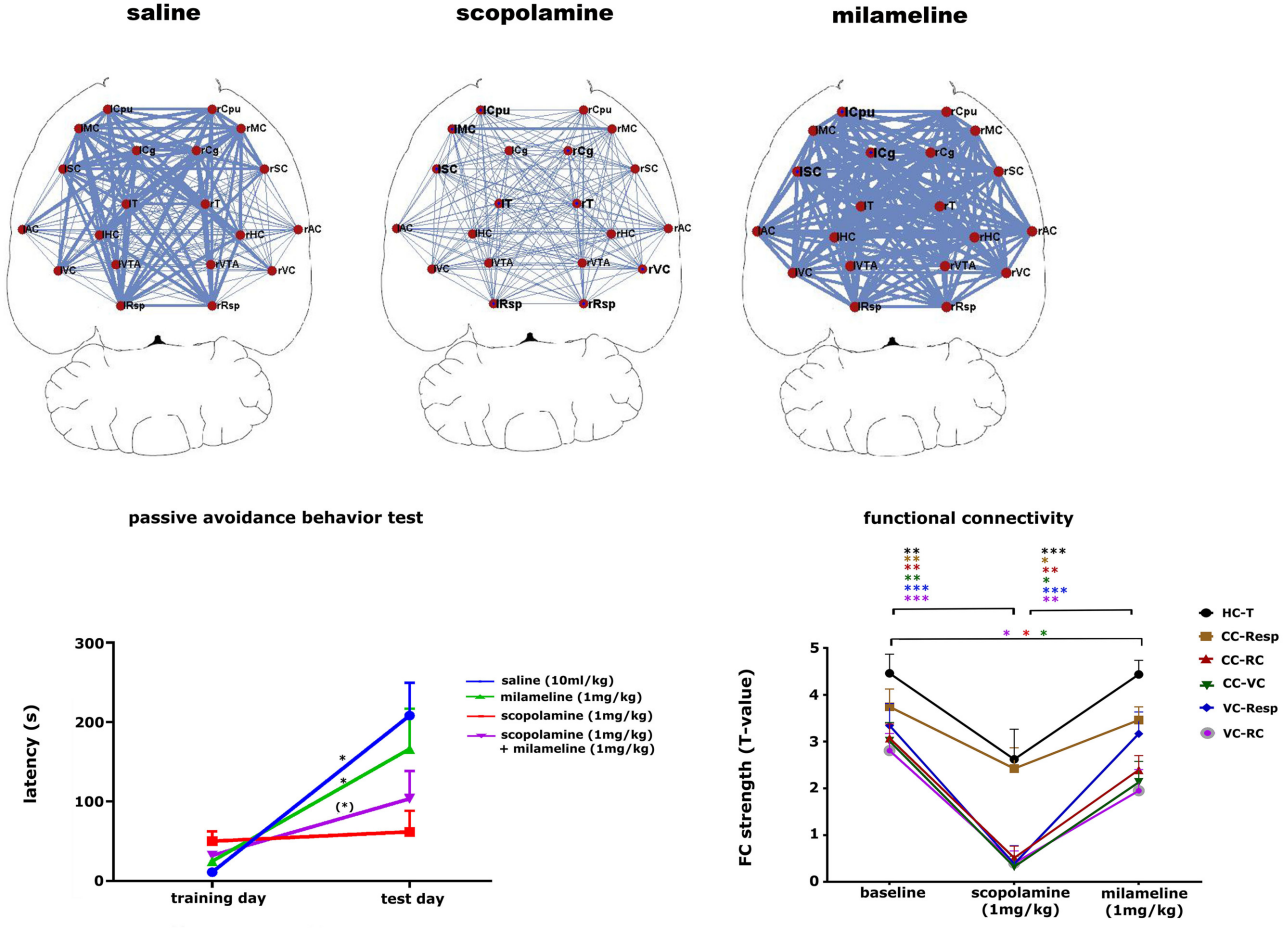
**Pharmacological modulation of the cholinergic system in mice.** The upper panel shows that scopolamine (1 mg/kg, s.c.) decreases FC and milameline (1 mg/kg, s.c.) increases FC compared to saline-treated (10 ml/kg, s.c.) animals. FC is shown as a ball-and-stick representation, with the thickness of the lines proportional to the strength of FC between each pair of brain regions. L, left; R, right; Cpu, caudate putamen; MC, motor cortex; Cg, cingulate cortex; SC, somatosensory cortex; T, thalamus; AC, auditory cortex; HC, hippocampus; VC, visual cortex; VTA, ventral tegmental area; Resp, retrosplenial cortex. The left lower panel shows the results of the passive avoidance test for the saline (10 ml/kg), scopolamine (1 mg/kg), milameline (1 mg/kg) treated groups and for a group treated subsequently with scopolamine and milameline. ^∗∗^*p* < 0.05, ^∗^*p* < 0.1. The graph shows that scopolamine impairs contextual memory, while the additional injection of milameline partially recovers scopolamine-induced memory impairments. The right lower panel shows a graph displaying the strength of FC at baseline, after the injection of scopolamine and after the subsequent injection of milameline. FC strength ± standard error are shown for FC between the hippocampus (HC) and thalamus (T), the cingulate cortex (CC), and retrosplenial cortex (Resp), the cingulate cortex and rhinal cortex (RC), the cingulate cortex and visual cortex (VC), the visual cortex and retrosplenial cortex and between the visual cortex and rhinal cortex. ^∗^*p* < 0.05, ^∗∗^*p* < 0.01, ^∗∗∗^*p* < 0.001. Scopolamine decreases all FC compared to baseline, and milameline recovers scopolamine-induced FC impairments completely or partially (figure reproduced with permission of Springer; Shah et al., 2015b).

The cholinergic system is important for learning and memory processes. Muscarinic cholinergic receptors (mAChR) are widely affected in AD, which might be tightly correlated with cognitive disabilities observed in those patients (Schneider et al., 2014). Pharmacological inhibition of cholinergic functioning with the mAcHR antagonist scopolamine leads to cognitive impairments that are similar to the behavioral characteristics observed in **dementia** (Klinkenberg and Blokland, 2010). The possibility of non-invasively detecting alterations of the cholinergic system in mice might greatly improve early diagnosis and treatment strategies in AD mouse models and eventually in the clinic. A recent study showed how phMRI and rsfMRI can be used as tools to detect alterations in the cholinergic system (Shah et al., 2015a). This study showed that scopolamine induced a dose-dependent effect on FC in brain regions with abundant mAChRs and are known to be involved in cognitive functions (**Figure 7**). Moreover, some FC deficits elicited by scopolamine could be completely recovered by administering a mAChR agonist milameline, while other FC deficits were not completely recovered. This result was consistent with the merely partial recovery of scopolamine-induced contextual memory deficits by milameline. This study showed how phMRI and rsfMRI can possibly be used as a non-invasive indicator of alterations in neurotransmitter systems induced by pathology or treatment.

Finally, the pharmacologically induced model that has been studied most extensively by fMRI, phMRI, and rsfMRI is **addiction**. The following exemplary addiction studies will illustrate the power and biological versatility of different fMRI techniques. First of all, fMRI can be used to pinpoint the neural substrate for addiction. For example, acute nicotine administration potentiates the brain reward function and enhances motor and cognitive function. This coincides with an increased BOLD signal in brain areas implicated in reward signaling (Johnson et al., 2013) (i.e., the striato-thalamo-orbitofrontal circuit, which plays a role in compulsive drug intake, and in the insular cortex, which contributes to craving and relapse) (Bruijnzeel et al., 2014). Second, including transgenic mouse models in fMRI experiments could be useful in explaining the contribution of certain receptor types in altered behavior induced by a drug. For example, acute nicotine injection results in increased brain activation in all cortical and subcortical regions of nicotine-naïve mice, which is not observed in knockout mice for the β2-containing nicotinic receptor. This nicotine injection triggered change in activation pattern can explain observed behavioral effects such as altered spatial learning, conflict solving etc., (Suarez et al., 2009).

Third, fMRI allows for the investigation of factors that modulate addictive behaviors and their neural substrates. For example, a differential sensitivity to cocaine is seen in female rats not only as a result of hormonal changes during/throughout the estrous cycle, but also in association with changes in sexual receptivity and presence of pups (Febo et al., 2011; Caffrey and Febo, 2014). FC analyses show connectivity effects in the brain which depend on the amount of time that has passed since the previous dosage of the drug, which implies that the same dose of nicotine might have a different impact on the brain depending on the time elapsed from the previous exposure (Huang et al., 2015). Finally, long term effects of addiction are extensively studied. FC analysis provided evidence of plasticity in addicted animals learned to self-administer cocaine, consistent with results in human drug addicts (Lu et al., 2014).

### (rs)fMRI Studies in Lesion Models and Transgenic Models

Both **stroke** and **neurological trauma (brain trauma and peripheral nerve injury)** are clear examples of pathologies where severe neurological damage occurs. St-fMRI and rsfMRI can detect the resulting loss of functionality (Pawela et al., 2010; Yao et al., 2012; Niskanen et al., 2013; Stephenson et al., 2013; Li et al., 2014; Shih et al., 2014) and connectivity on a functional level (van Meer et al., 2010a; Baliki et al., 2014; Mishra et al., 2014). Interestingly, in most cases this loss of functionality is partially recovered depending on the lesion severity (Niskanen et al., 2013; Shih et al., 2014). The process of recovery which relates to neuroplasticity and network reorganization, can be monitored using the same techniques (van Meer et al., 2010b, 2012; Li et al., 2014). Finally, rsfMRI can be used as a read-out for treatment efficacy resulting in different strategies to ameliorate recovery (Wang et al., 2012; Suzuki et al., 2013).

**FIGURE 8:**
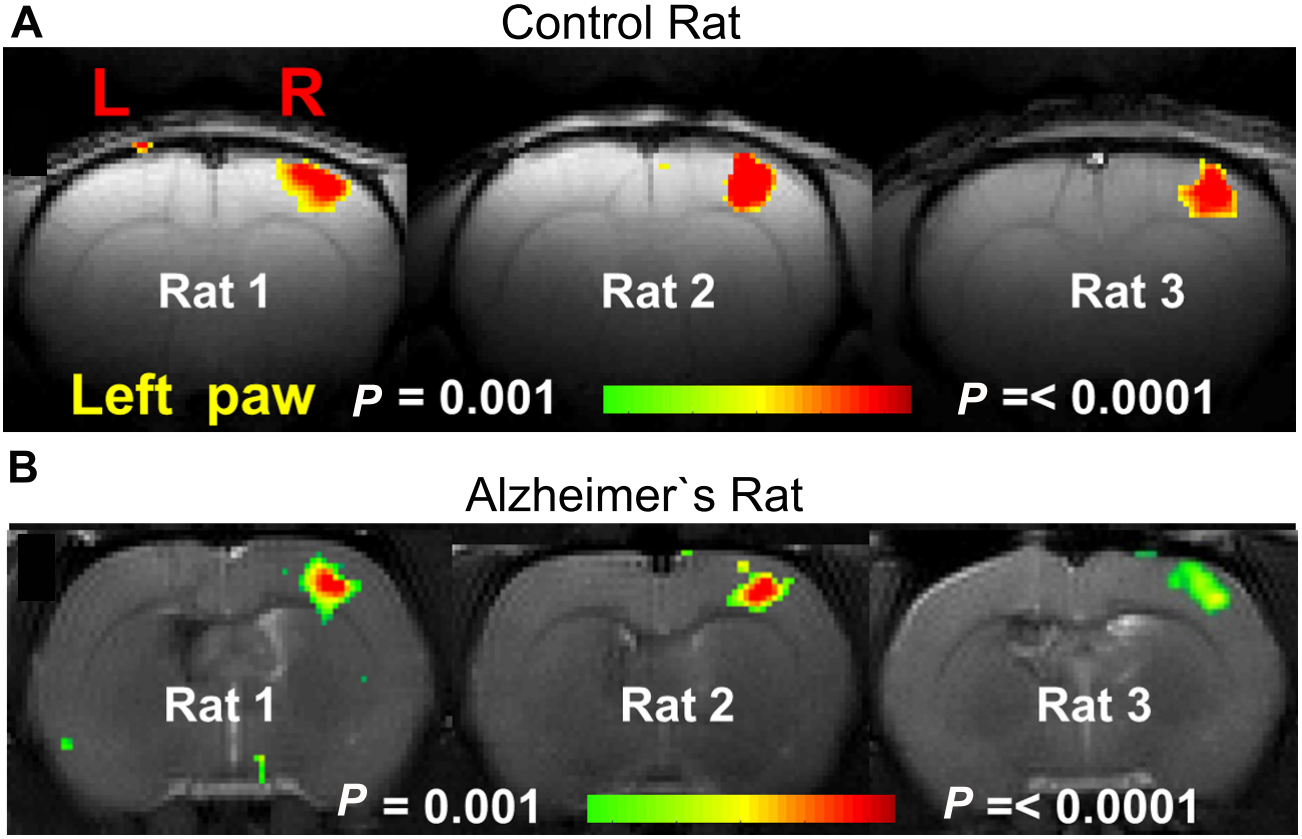
**BOLD activation maps during contralateral forepaw stimulation in **(A)** control rat and **(B)** AD rat.** Rats were anesthetized with α-chloralose and ventilated. Much smaller BOLD activation patterns were observed in the somatosensory forelimb cortex (S1FL) in the AD rats compared to the control rats [Figure based on the results shown in with permission of the author (Sanganahalli et al., 2013)].

**FIGURE 9:**
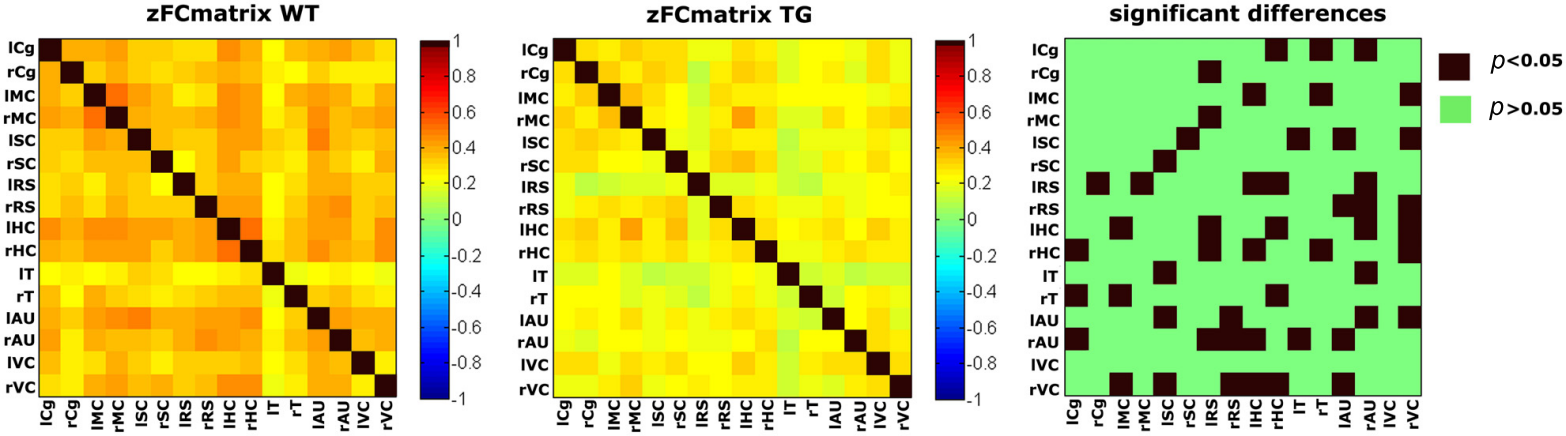
**Functional connectivity matrices under medetomidine anesthesia, acquired in aged matched wild type (wt) and transgenic AD mice (TG) with an average age of 18.9 months.** Statistical comparison (right) clearly shows a widespread decrease in FC in the diseased animals (Shah et al., 2013).

A lot of preclinical neuroscience work is performed on transgenic rodent models for **neurodegenerative diseases** such as: AD, PD, and HD, which are characterized by deposition of misfolded proteins (proteinopathies) in the brain. In AD and HD, the presence of amyloid plaques and huntingtin, respectively, are hypothesized to affect cortical functioning as shown by diminished fMRI responses to sensory stimuli (Lewandowski et al., 2013; Sanganahalli et al., 2013; see **Figure 8** for an example). Moreover, entire neuronal networks seem affected (Liu et al., 2014), as shown by altered functional connectivity during rsfMRI (Shah et al., 2013; Ferris et al., 2014; **Figure 9**) even in early disease stages before the proteinopathy establishes (Grandjean et al., 2014b). Compared with wild-type mice, FC deficits are also reported in both adult and old apoE4 and apoE-KO mice. This finding could be related to the fact that the risk of developing neurodegeneration is dependent on the present cholesterol-transporter apolipoprotein 𝜀 (APOE) genotype (Zerbi et al., 2014).

Functional magnetic resonance imaging tools are equally suited to study which factors, for example environmental enrichment, stressors, etc., could interfere with neuronal network modulation over time (Little et al., 2012). Similarly, the effects of exercise on ameliorating the affected FC network in Parkinsonian rats was studied (Wang et al., 2015). Clearly both st-fMRI and rsfMRI could be used as read-outs for the efficacy of new therapies in neurodegenerative diseases.

### Translation of Application of fMRI Techniques in Pharmacological Research from Rodents to Humans

The non-invasive nature of fMRI renders the technique valuable in terms of translation to the clinic. Though the results in animal models can be translated to the pathology in humans, daily application into the clinic is still hampered by the required sophisticated data analysis. Nevertheless, in clinical research, both st-fMRI and rsfMRI protocols are well established, but phMRI following an acute pharmacological compound injection in the magnet, to characterize the target regions of this compound, is used to a lesser extent. For st-fMRI, an important difference is the type of stimulus used since in rodents typically sensory stimulation is used while in humans also other (complex cognitive) tasks could be used as stimulus.

On the other hand, the fact that rsfMRI requires no action or cooperation of the patient – which is sometimes not feasible under pathological conditions –encouraged clinical research in measuring the brain’s resting state clearly before it was even applied on a preclinical level.

It is important to notice that most of the clinical FC research is focusing on the default mode network. A DMN-like network, covering to a certain extent the same anatomical regions, was also observed in rodent models making the technique even more translational. However, the majority of preclinical studies apply a whole brain approach making use of the advantage of rsfMRI to have information on all brain networks obtained within a single short MRI scan.

Some important differences have to be taken into account when comparing acquisition of rodent fMRI with human fMRI. Most small animal MRI systems operate at 4.7–9.4 Tesla and even 11.4T field strengths. Although very recently high field human MRI systems are installed in clinical research centers, typically lower field strengths are used (1.5–3T) in the clinic. The higher field strength clearly results in a higher signal-to-noise ratio, which enables a higher spatial resolution as needed for rodents. On the other hand, artifacts are typically more prominent. For example in the head, which is comprised of multiple compartments with different physico-chemical properties, artifacts can be generated due to susceptibility and different relaxation properties at the higher field strength resulting in image distortions at the level of the ear cavities (Bernstein et al., 2006). As discussed in detail most animal experiments are performed under anesthesia, enabling complete immobilization of the subject, minimizing motion artifacts which can even be improved by mechanical ventilation of the animal.

For most of the applications reviewed above, convergent studies are available in humans demonstrating the clear translational quality of fMRI research. As clinical research is beyond the scope of this review, we only provide some representative illustrations and related references of key reviews in clinical research.

As outlined above, fMRI is capable of unraveling the neuronal substrate of **schizophrenia.** Similarly in humans, functional neuroimaging helped to unravel the neuronal basis of the positive, negative and cognitive symptoms of the disease. Moreover, fMRI studies assisted in developing therapeutic strategies and defining promising targets. For example, both the reported animal work and human studies pinpointed the importance of NMDA receptors in schizophrenia (Gruber et al., 2014).

fMRI can also be used to localize and monitor **epileptic** seizures, similarly as pharmacologically induced seizures in rodents. Additionally, on a clinical level, both st-fMRI and rsfMRI can be used to explore how seizures modulate the brain and its organization (Chaudhary and Duncan, 2014).

In MDD, changed brain activation (st-fMRI) and FC anomalies were reported both in humans and animal models (Dutta et al., 2014; Kerestes et al., 2014). Moreover clinical trials have also examined the immediate or delayed effects of antidepressants on resting state networks (Dichter et al., 2014).

In the past two decades, imaging studies of drug **addiction** have demonstrated functional brain abnormalities by studying drug-addicted human populations (Parvaz et al., 2011) and rodents. Moreover, acute brain response to addictive substances are studied in humans (Hommer et al., 2011).

For **stroke** imaging there is, both in preclinical and clinical research, a clear research-focus on network reorganization after the insult and during recovery. Moreover fMRI can contribute to improve prognostic ability and the development of therapeutic interventions (Carter et al., 2012). Although in animals both rsfMRI and st-fMRI has been performed, most studies in stroke patients report rsfMRI data (Veldsman et al., 2015). Evidently, acute modulations of the brain immediately after the insult are more convenient to study in rodents than in humans.

For **neurodegenerative** diseases, st-fMRI upon a conscious task can be highly compromised by the mental status of the patients. Studies in cognitively healthy individuals with brain amyloidosis or genetic risk factors for AD have shown functional connectivity abnormalities in preclinical disease stages (Pievani et al., 2014) in convergence with the findings of Grandjean et al. (2014b) in mice. An important difference between animal models and humans is that human neurodegenerative diseases (e.g., AD) are complex diseases manifesting different features. Using animal models, one can differentiate pathological features in separate models which show one key aspect of the disease (e.g., tau and amyloid pathology in AD). In this way, the observed differences in humans may be linked to a certain hallmark of the disease.

## Future Perspectives

Since its first implementation, the field of fMRI has grown exponentially. st-fMRI, rsfMRI, and phMRI have been used intensively to not only characterize the functional properties and organization of healthy brains but also to evaluate pathogenesis and inspect treatment efficacy both in humans and animal models. In addition, many fascinating advanced processing techniques have become available that allow researchers to comprehend complex network topology and infer effective connectivity.

Modern brain mapping techniques, such as rsfMRI, produce increasingly large datasets of functional connection patterns underlying the large-scale functional organization of the brain^5^

Concurrent technological advances are generating similarly large connection datasets in biological, technological, social, and other scientific fields. Attempts to characterize these datasets have, over the last decade, led to the emergence of a new, multidisciplinary approach to the study of complex systems. This approach, known as **complex network analysis**, describes important properties of complex systems by quantifying topologies of their respective network representations (Rubinov and Sporns, 2010).

Secondly, new developments in **brain circuit modulations** such as deep brain stimulation (Younce et al., 2014; Lai et al., 2015) and the more targeted optogenetic modulations of neuronal populations (ofMRI) can be used to study brain networks. Brain circuit elements can be selectively triggered with temporal precision while the resulting network response is monitored non-invasively with high spatial and temporal accuracy (Lee, 2011).

Most resting-state fMRI investigations were based upon static descriptions of FC. However, since the brain must dynamically integrate, coordinate, and respond to internal and external stimuli across multiple time scales, recent studies have begun to study the dynamics of FC over time. Emerging evidence suggests that using **dynamic rsfMRI,** FC changes in macroscopic neural activity patterns can be discovered which may underlie critical aspects of cognition and behavior. Nevertheless limitations with regard to analysis and interpretation should be taken into account (Hutchison et al., 2013).

Though the focus of this review lies on BOLD-based neuronal activity measurements, we want to emphasize that **ASL,** which was briefly introduced earlier in this review, provides a non-invasive, absolute quantification of CBF both at rest and during task/drug activation. These CBF measurement are very stable over time, and as such ASL allows a relatively straightforward physiological interpretation of drug-induced changes in neuronal activation. ASL studies which follow CBF changes in response to a specific stimulus/task or pharmacological modulation are available for human research [see review, (Wang et al., 2011)]. These techniques can be translated from clinical to rodent research to detect either the central effects of a drug or the change in neuronal response following drug administration. One recently published study, presents data from 1400 rats following a standardized ASL-phMRI protocol using different known compounds (Bruns et al., 2015). Based on these data, a new method was proposed to quantitatively characterize new psychotropics in which typical and differential activation profiles after antipsychotic, antidepressant, and anxiolytic drug injection could be defined. Similar approaches could be used for BOLD fMRI as well with interesting opportunities for future pharmacological research. The acquired ‘typical activation patterns‘ can be used to classify drug induced brain activations of unknown psychotropics which further facilitates biological understanding and decision making in drugdiscovery (Bruns et al., 2015).

The current developments in measuring CBF by pseudo-continuous ASL on clinical scanners (Alsop et al., 2014) have dramatically improved the sensitivity and essential high temporal resolution of perfusion imaging to allow the detection of CBF based functional connectivity changes (Chuang et al., 2008; Fernandez-Seara et al., 2011). However, similar ASL acquisition protocols on preclinical scanners still need to be optimized to provide stable CBF measurements with a high spatial and temporal resolution to allow similar FC-analysis. This would create new avenues in pharmacological rodent research coming from the combination of BOLD and ASL-based MRI. Combining both BOLD and ASL-based rsfMRI in rodents would allow to perform joint FC-analysis (Jann et al., 2015) to characterize the spatiotemporal brain networks within phMRI studies. On the other hand, from the BOLD and ASL-data, it is possible to extract a third read-out of neuronal activity, being the cerebral metabolic rate of oxygen (CMRO2), which is less sensitive to vascular dynamics. Similar to BOLD and ASL experiments, one can follow drug induced changes of CMRO2 within a rsfMRI, st-fMRI, or phMRI design.

In conclusion, fMRI based on the BOLD response can be used in a wide range of different applications. Recently, sensory and pharmacologically induced fMRI were extended with fMRI measured during rest. The interest in rsfMRI is growing, resulting in an increasing number of methods to analyze and interpret the data. This review clearly shows the potential of MRI to study neuro-modulation, particularly induced by pharmacological agents. Functional MRI techniques enable researchers to obtain a vast amount of information in a relatively short amount of time compared to other imaging techniques. Moreover, we are at the beginning of fMRI’s application to preclinical treatment testing, especially rsfMRI.

Another important feature which makes fMRI a unique and highly exceptional method compared to other ‘brain targeting tools’ such as electrophysiology, is its translational character. Indeed, many similarities have been reported between human and small animal findings. However, in general, animal studies are lagging behind studies in humans when it comes down to assessing the effects of specific pathologies on functional characteristics of the brain. With this review, we hope to have convinced and maybe even inspired neuroscientists to further exploit fMRI and its many applications in (pre)clinical setting.

## Conflict of Interest Statement

The authors declare that the research was conducted in the absence of any commercial or financial relationships that could be construed as a potential conflict of interest.

## Footnotes

1 http://www.fil.ion.ucl.ac.uk/spm/software/spm8

2 http://www.fmrib.ox.ac.uk/fsl.

3 http://brainatlas.mbi.ufl.edu/

4 http://scalablebrainatlas.incf.org/

5 https://www.humanbrainproject.eu/.

## ABBREVIATIONS

5-HT: serotonin
5-HT1A-R: serotonin 1A receptor
AD: Alzheimer’s disease
ALFF: amplitude of low frequency fluctuations
ASL: arterial spin labeling
BOLD: blood oxygenation level dependent
CBF: cerebral blood flow
CBV: cerebral blood volume
CMRO2: cerebral metabolic rate of oxygen consumption
CT: computed tomography
DCE: dynamic contrast enhanced
DSC: dynamic susceptibility contrast
EEG: electroencephalography
EPI: echo planar imaging
FC: functional connectivity
FDG: fluoro-deoxy-glucose
fMRI: functional MRI
FSL: FMRIB software library
GABA: gamma amino-butyric acid
GRASE: gradient- and spin-echo
GS: global signal
HD: Huntington’s disease
HDR: hemodynamic response
ICA: independent component analysis
LFP: local field potential
mAChR: muscarinic Acetylcholine receptor
MEG: magnetoencephalography
MRI: magnetic resonance imaging
NIRS: near-infrared spectroscopy
NMDA: *N*-methyl-D-aspartate
ofMRI: optogenetic fMRI
pCO_2_: partial pressure of CO_2_
PD: Parkinson’s disease
PET: positron emission tomography
phMRI: pharmacological MRI
ROI: region-of-interest
rsfMRI: resting state fMRI
SPM: statistical parametric mapping
st-fMRI: stimulus-evoked fMRI
SSRI: selective serotonin re-uptake inhibitors.
